# A Type-Aware Approach to Message Clustering for Protocol Reverse Engineering

**DOI:** 10.3390/s19030716

**Published:** 2019-02-10

**Authors:** Xin Luo, Dan Chen, Yongjun Wang, Peidai Xie

**Affiliations:** 1 College of Computer, National University of Defense Technology, Changsha 410073, China; luoxin13@nudt.edu.cn (X.L.); xpd2002@126.com (P.X.); 2 School of Cyberspace Security, Hangzhou Dianzi University, Hangzhou 310018, China; dan.chen@ieee.org

**Keywords:** message clustering, protocol reverse engineering, Internet of Things, information security

## Abstract

Protocol Reverse Engineering (PRE) is crucial for information security of Internet-of-Things (IoT), and message clustering determines the effectiveness of PRE. However, the quality of services still lags behind the strict requirement of IoT applications as the results of message clustering are often coarse-grained with the intrinsic type information hidden in messages largely ignored. Aiming at this problem, this study proposes a type-aware approach to message clustering guided by type information. The approach regards a message as a combination of n-grams, and it employs the Latent Dirichlet Allocation (LDA) model to characterize messages with types and n-grams via inferring the type distribution of each message. The type distribution is finally used to measure the similarity of messages. According to this similarity, the approach clusters messages and further extracts message formats. Experimental results of the approach against Netzob in terms of a number of protocols indicate that the correctness and conciseness can be significantly improved, e.g., figures 43.86% and 3.87%, respectively for the CoAP protocol.

## 1. Introduction

Edge computing and artificial intelligence have driven the development of the Internet of Things (IoT) [1,2,3,4]. In information security of the IoT, Protocol Reverse Engineering (PRE) has long been pursued, which is the process of extracting parameters, formats, and semantics of protocols without access to formal specifications [5]. Protocol specifications are crucial for protocol evaluation, protocol reusing, malware detection, etc. To extract the specification of a protocol from network traffic, a typical routine of PRE includes message clustering, message formats extraction, and protocol state machines extraction. Amongst them, the extraction of message formats and protocol state machines depends on the capability to precisely cluster messages.

Similar messages are believed to belong to the same type. This study defines type information as the probability that a message belongs to some type. Message clustering applies clustering algorithms to group similar messages. These messages often share the same format. For instance, in FTP protocol implementations, messages used to log in servers are clustered as a group, which are formatted as “USER username \r\n”. To cluster messages, approaches based on message character sequence alignment have been widely used. However, these approaches are limited to finding the similarity of message bytes data. Directly applying sequence alignment approaches to message clustering will inevitably ignore the latent type information in messages. This will lead to coarse-grained clustering results.

In theory, as high-level semantic information of messages, type information is intrinsically within messages. Since message clustering puts together messages of the same type, its results will be improved if type information is properly used. However, it is not easy to confirm types of messages with limited prior knowledge. Furthermore, approaches need to properly represent type information and use it to guide message clustering.

Some existing approaches [6,7] employ topic generative models to extract message formats. They characterize messages with keywords and n-grams via inferring keyword distributions for each message. However, such approaches still focus on basic literal message data, with type information ignored. Two research challenges remain before type information can be exploited to guide message clustering:

1. Practically, it is no cinch to properly represent type information. How to express this information as model components still remains to be solved.
2. In theory, the results of message clustering will be improved with the help of type information. However, it is needed to figure out how to apply type information into message clustering.

To tackle these challenges, an appropriate solution should be able to (1) properly express type information as model components, and (2) ensure that the extracted type information can be effectively used to guide message clustering. This study develops a type-aware approach to message clustering. The approach employs n-gram model to represent protocol messages. It regards a message as a combination of n-grams. It also incorporates Latent Dirichlet Allocation (LDA) model to characterize messages with types and n-grams via inferring type distributions for each message. The type information in the approach is type distributions of LDA model. Such distribution is used as distance metric in message clustering. Guided by type information, messages are properly clustered according to their types.

To evaluate the effectiveness of this approach, a number of experiments were carried out. During evaluation, the message clustering approach in Netzob was first replaced by the proposed approach. Then, experiments were conducted to choose optimal parameters for the approach. Finally, this study made a comparison between two approaches, with respect to the correctness and conciseness.

The main contributions of this study are as follows:

1. A solution has been proposed to properly represent type information. This enables a fine-grained message clustering which takes into consideration type information or the higher-level semantic information.
2. An approach to message clustering is developed. It uses a new similarity metric to guide message clustering, which is based on type information instead of literal alignment results.

## 2. Related Work

Numerous attempts have been made to reverse the formats of messages sent by a network application, which is crucial for information security of IoT [8,9]. Studies undertaken for this purpose focus on (1) approaches based on network traces [10] and (2) approaches based on execution traces [11]. In the context of IoT, with limited access to binary implementations, it is practically easier to access network traces and use them to reverse message formats. The most salient works along this direction are introduced as follows.

Beddoe et al. [10] proposed PI Project to use sequence alignment to compare similar messages and detect the fields. However, PI used alignment scores to cluster messages and users must analyze the clustering tree to extract message formats. Following PI Project, Bossert et al. [12] proposed Netzob to characterize botnets’ communication, in which they inferred message formats and protocol state machines. Netzob relied much on expert knowledge. During inference, users must provide observed delimiters and key fields to cluster messages. In [13], the authors used a segment-based alignment approach to extract packet structures, which worked around the limitations of global alignment. But their approach to message clustering was based on binary distance measure, which was limited to literal bytes data.

To exploit deeper information of messages, some language models have been employed to cluster messages or extract message formats. Wang et al. [14] proposed to employ a Latent Dirichlet Allocation (LDA) model to describe the relationship among messages, keywords, and message n-grams. However, their work was still limited to the literal message data and the output of LDA model needed post processing before being available. In [15], the nonparametric Bayesian statistical model was modified to identify field boundaries.

Amongst above approaches, sequence alignment or other measures have been used as similarity of messages. Message clustering in those approaches only used observed literal information, with semantic information hidden in messages ignored. This led to coarse-grained results of message clustering. Besides, these approaches often needed extra expert knowledge or manual work, which limited the automation of these approaches. To give a deeper insight into messages data, the proposed approach takes type information into consideration and has the following major concerns: (1) to use the higher-level semantic information for fine-grained message clustering, and (2) to improve the automation of existing approaches.

## 3. Latent Dirichlet Allocation Model and Its Inference

It is desirable to find an approach to describing type information of protocol messages. This study tries to employ topic generative models to characterize protocol messages with their type information. To explain how this model works, this section first recaps basic ideas of LDA and how LDA generates documents over some topics. Then, the approach to model inference is demonstrated.

### 3.1. How LDA Works

Topic generative models can be used to analyze the evolution of unobserved topics of a collection of documents. The Latent Dirichlet Allocation (LDA) model [16] is a typical topic generative model used to discover the abstract “topics” that occur in a collection of documents. Given that a document is about some specific topic, LDA assumes that specific words will appear in this document more or less frequently. Therefore, LDA can discover topics for each document based on the statistics of words in these documents.

**Figure 1:**
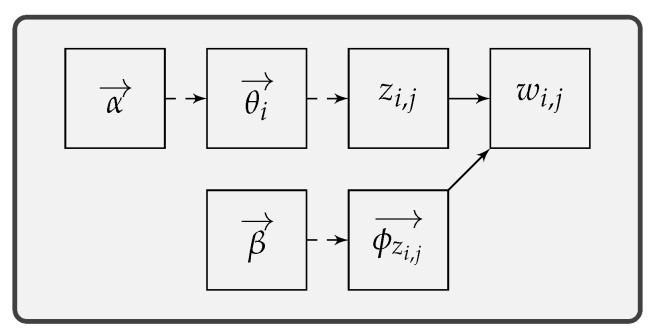
The generation of a document under LDA.

Figure 1 illustrates how LDA generates a document. In LDA, documents are represented as mixtures over topics, with each topic characterized by a distribution over words. In this way, LDA can be simplified as two distributions: (1) $\vec{\alpha }$ for per-document topics and (2) $\vec{\beta }$ for per-topic words. Each distribution is controlled by its Dirichlet prior parameter and comes with its conjugate distribution. LDA assumes a document to be generated in the following steps:

1. Sampling topic distribution $\vec{\theta_{i}}$ from $\vec{\alpha }$, i.e., the topic distribution for the *i*-th document;
2. Sampling topic $z_{i,j}$ from $\vec{\theta_{i}}$, i.e., the topic for the *j*-th word in the *i*-th document;
3. Sampling word distribution $\vec{\phi_{z_{i,j}}}$ from $\vec{\beta }$, i.e., the word distribution for topic $z_{i,j}$;
4. Sampling word $w_{i,j}$ from $\vec{\phi_{z_{i,j}}}$, i.e., the *j*-th word in the *i*-th document;
5. Repeating 2, 3, and 4 to sample all the words for the *i*-th document.

With this assumption, documents can be characterized with topics and words via inferring the topic distribution of each document. Since the statistics of words reflect topics, the topic distribution can be inferred by observing these statistics.

For protocols, it is similar to generate a message. A protocol message can be viewed as a mixture over types. Message n-grams appear more or less frequently in specific types of messages. This makes it possible to infer type distribution via statistics of message n-grams. Considering such resemblance between protocol messages and documents, this study employs LDA to characterize messages with message types and n-grams.

### 3.2. Inference of LDA

In this study, the type information is defined as the type distribution in LDA. The inference of type distributions in LDA is often approximated by the *Markov Chain Monte Carlo* algorithm (MCMC). This approach uses Gibbs Sampling, a typical MCMC algorithm, to extract type distributions for each message.

Assuming that there are *M* messages and *T* message types, the n-gram vectors and their corresponding message types are denoted as $\vec{w}=\left(\vec{w_{1}},\ldots ,\vec{w_{M}}\right)$ and $\vec{z}=\left(\vec{z_{1}},\ldots ,\vec{z_{M}}\right)$, where $\vec{w_{i}}$ denotes n-grams vector for the *i*-th message and $\vec{z_{i}}$ denotes its type. With all the *M* messages, p(w) denotes the probability that n-gram *w* appears in all the *M* messages, $p(z=z_{t})$ denotes the probability that the type of a message is $z_{t}$ and $p(w|z=z_{t})$ denotes the probability that n-gram *w* appears in messages that belong to type $z_{t}$. For all the messages of *T* types, Equation (1) illustrates how to calculate p(w).

(1) $$p\left(w\right)=\sum_{i=1}^{T}p\left(w|z=z_{i}\right)p\left(z=z_{i}\right),\;\;\sum_{i=1}^{T}p(z=z_{i})=1$$

With posterior occurrences of $\vec{w}$ already known, this approach aims at inferring the type distribution $p(\vec{z}|\vec{w})$, the probability distributions over types.

For specific variables, Gibbs sampling tries to update their values by iteratively sampling from the corresponding variable distribution. Therefore, in order to estimate $p(\vec{z}|\vec{w})$, this approach replaces each $z_{i}$ with a value sampled from $p(z_{i}|\vec{z_{\neg i}},\vec{w})$. When the model has converged, the approach will stop sampling and output the type distributions for each message.

## 4. Type-Aware Message Clustering

The output type distributions of LDA are regarded as type information of protocol messages. Such type information is used to develop the type-aware approach to message clustering. The proposed approach is a module in PRE frameworks. This study implements the proposed approach to improve existing PRE frameworks.

### 4.1. Overview of the Proposed Approach

**Figure 2:**
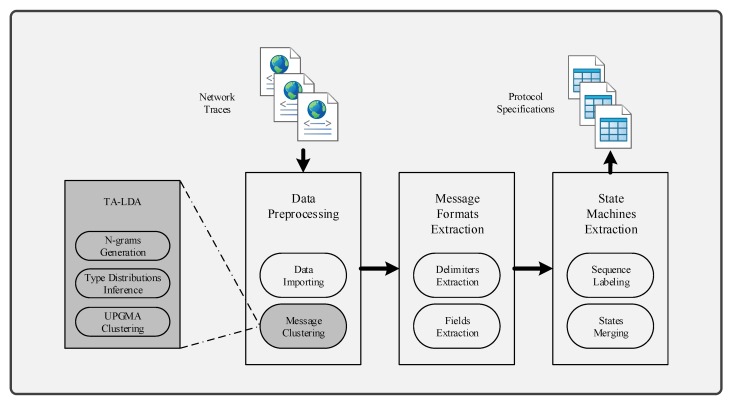
Overview of TA-LDA.

Figure 2 illustrates the overview of TA-LDA, i.e., a Type-Aware LDA based approach to message clustering. TA-LDA is developed as a module of PRE frameworks. A typical PRE framework should include at least three components: (1) data preprocessing, (2) message formats extraction, and (3) state machines extraction. This study implements a type-aware approach to message clustering, which is crucial for message formats extraction and state machines extraction.

The proposed approach takes protocol messages as input and outputs message clusters. It uses LDA to extract type distributions for each message, which are used as type information to cluster messages. First, before feeding into LDA, messages are tokenized using n-gram model. This splits messages into vectors of n-grams and tokenizes these vectors. Then, n-grams vectors are used as corpora to train the LDA model, i.e., inferring the type distributions for those messages. Next, the approach uses type distributions as distance vectors for each message. Finally, typical clustering algorithms are adopted to cluster messages according to the calculated distance vectors.

The following subsections detail how the approach extracts type information and uses it to cluster messages.

### 4.2. N-Grams Generation

In many existing PRE frameworks, basic message elements are bytes, which only contain literal information. The n-gram model has been widely used in many disciplines. Previous researches have employed n-gram model to describe messages. Following these approaches, this study also uses n-grams as basic message elements.

An n-gram is *n* consecutive bytes. To give an example, for the sequence “HTTP 1.0\r\n 200 OK”, every four bytes can form a four-gram. Therefore, four-grams for this sequence can be “HTTP”, “TTP”, “TP 1”, “P 1.”, “ 1.0”, …, “00 O”, “0 OK”. In this way, a sequence of bytes can be divided into basic n-grams.

However, many n-grams might not contribute to type information. To filter these extra n-grams, this study employs TF-IDF algorithm [17] to exclude unimportant n-grams.

Given a collection of messages, *M*, Equations (2) and (3) define how to calculate TF and IDF scores, where $n_{i,j}$ denotes the times that the *i*-th n-gram appears in the *j*-th message and $t_{i}$ denotes the *i*-th n-gram. Equation (4) defines how to calculate TF-IDF scores.

(2) $$TF_{i,j}=\frac{n_{i,j}}{|\vec{m_{j}}|}$$

(3) $$IDF_{i}=lg\frac{\left|M\right|}{1+|\{d\in M|t_{i}\in d\}|}$$

(4) $$TFIDF_{i,j}=TF_{i,j}\times IDF_{i}$$

With TF-IDF algorithm, this approach only selects n-grams that can better describe the type information for each message.

### 4.3. Type Distributions Inference

To infer type distributions for each message, this approach employs Gibbs Sampling algorithm to infer parameters of LDA model, which include the target type distributions. Algorithm 1 demonstrates the whole process.

In Algorithm 1, $n_{m}^{\left(t\right)}$ is the times that an n-gram for message *m* is assigned to type *t* and $n_{t}^{\left(g\right)}$ is the times that n-gram *g* is assigned to type *t*. During each iteration, the type for each n-gram is resampled from the output distribution conditioned on the other variables. When the model has converged, the type distributions for each messages are accordingly output, which are type information used in this study.
**Algorithm 1:** LDA with Gibbs Sampling
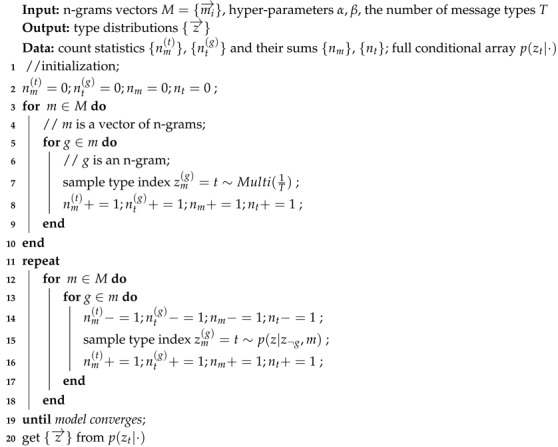


### 4.4. Message Clustering Based on Type Information

In traditional sequence alignment based approaches, alignment scores are used as similarity of messages. Semantics, e.g., keywords, can also be used as distance metric of messages. These distance metrics can perform well. However, the aim of message clustering is actually to cluster messages over the same types. This indicates the importance of type information.

This approach extracts type distributions for each message. For each message, the distribution is a probability array marked as $p\left(t|m\right)=[p\left(t_{1}|m\right),p\left(t_{2}|m\right),\ldots ,p\left(t_{k}|m\right)]$, where $p(t_{k}|m)$ represents the probability that message *m* is of type $t_{k}$. For messages of the same type, their probability arrays should be similar. Thus, we defined $D(m_{i},m_{j})$, the distance between message $m_{i}$ and message $m_{j}$, as shown in Equation (5).

(5) $$D\left(m_{i},m_{j}\right)=\sum_{k=1}^{T}|p\left(t_{k}|m_{i}\right)-p\left(t_{k}|m_{j}\right)|$$

On the basis of such distance metric, clustering algorithms are further employed, e.g., UPGMA [18], Information Bottleneck [19], K-Means [20] and etc. This approach simply uses UPGMA to cluster messages according to their type information, i.e., type distributions from the LDA model.

## 5. Experiments and Results

Experiments were carried out to evaluate the effectiveness of this approach. Considering that most existing frameworks are not publicly available, this paper selects Netzob, one of the open-source PRE frameworks, as a basic framework. In Netzob, there have been some approaches for message clustering, e.g., clusterByAlignment, clusterByKeyField, clusterbyApplicativeData and etc. This study developed an approach clusterByLDA to replace the original clustering approaches.

This section first introduces the evaluation criteria. Then data collection is briefly covered. Next, it is demonstrated how parameters can affect model performance. Finally, the improvements against Netzob are illustrated.

### 5.1. Evaluation Criteria

To compare with Netzob, this study used Conciseness and Correctness as evaluation criteria. Conciseness indicates whether true formats can be described in as few models as possible, which means there should not be extra inferred models for a true format. Differently, correctness indicates whether inferred models are valid, which means that messages in a cluster should actually share the same true format.

Let *M* be the set of messages, $F_{infer}$ be inferred formats and $F_{true}$ be true formats. Two functions, $I:\;M\to F_{infer}$ and $T:\;M\to F_{true}$ are defined. I(m) denotes the inferred format for message *m* and T(m) denotes its true format. Equation (6) defines the function $N_{correct}:F_{true}\to N$.

(6) $$N_{correct}\left(f\right)=\left|\left\{T\left(m\right)\;\;\forall m\in M\;\mathrm{such}\;\mathrm{that}\;I\left(m\right)=f\;\right\}\right|$$

Equation (7) defines the function $N_{concise}:F_{infer}\to N$.

(7) $$N_{concise}\left(f\right)=\left|\left\{I\left(m\right)\;\;\forall m\in M\;\mathrm{such}\;\mathrm{that}\;T\left(m\right)=f\;\right\}\right|$$

According to functions $N_{correct}$ and $N_{concise}$, Equations (8) and (9) calculate correctness and conciseness, respectively.

(8) $$Correctness=p(N_{correct}=1)\times 100$$

(9) $$Conciseness=p(N_{concise}=1)\times 100$$

### 5.2. Data Collection

This approach aims at improving message clustering in the reverse engineering of IoT protocols. This study analyzed some typical IoT application protocols. Amongst them, CoAP (Constrained Application Protocol) is designed for those micro devices and is an application protocol based on UDP. XMPP (Extensible Messaging and Presence Protocol) is a communication protocol for message-oriented middle-ware. CoAP and XMPP were used as our target protocols. Considering the comparison for other protocols, FTP was also used to experiment.

For CoAP, CoAPthon [21], an open-source implementation, was employed to simulate communications and messages were collected from the communication channel. For XMPP, since it is defined in XML format, messages data were generated by filling values for specific keys. For FTP, public datasets [22] were adopted. With data collected, this approach first preprocessed them. For sniffed PCAP data, WireShark [23] was employed to filter the noisy messages and data beyond the predefined length were cut off. For generated data, no preprocessing was done.

### 5.3. Parameter Tuning

Parameters can affect the performance of a model. Perplexity is widely used to measure the generalizability of the model. Equation (10) defines perplexity, where $p(w_{m})$ is the probability that n-grams of the m-th message occur and $N_{m}$ is the number of n-grams in the m-th message.

(10) $$\begin{array}{c} Perplexity\left(D_{test}\right)=\exp\left\{-\frac{\sum_{m=1}^{M}\log p(w_{m})}{\sum_{m=1}^{M}N_{m}}\right\} \end{array}$$

**Figure 3:**
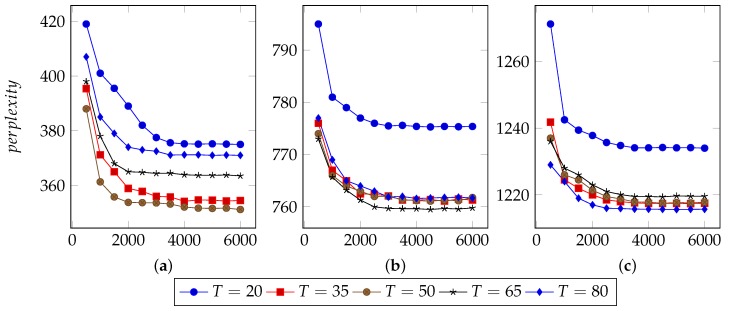
Selection of I for FTP. (**a**) Perplexity for model with 40% n-grams used; (**b**) Perplexity for model with 60% n-grams used; (**c**) Perplexity for model with 80% n-grams used.

**Figure 4:**
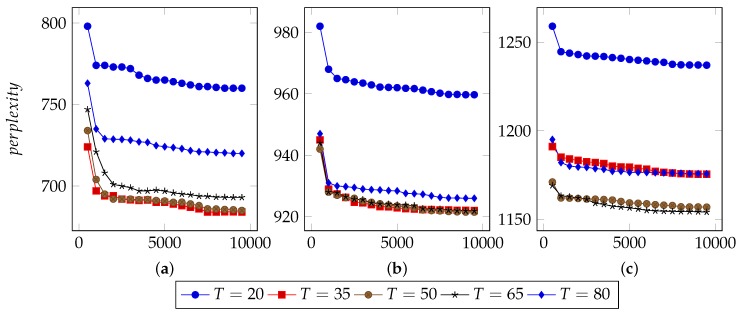
Selection of I for CoAP. (**a**) Perplexity for model with 40% n-grams used; (**b**) Perplexity for model with 60% n-grams used; (**c**) Perplexity for model with 80% n-grams used.

**Figure 5:**
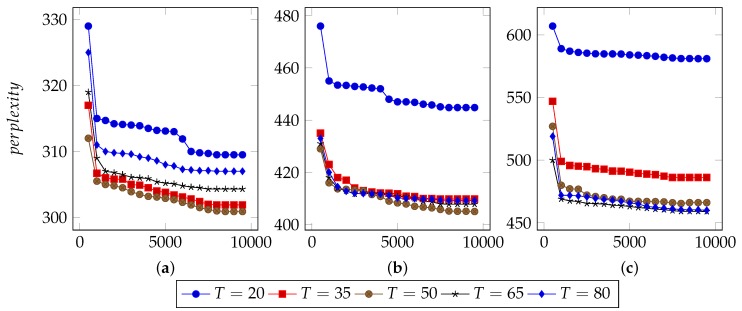
Selection of I for XMPP. (**a**) Perplexity for model with 40% n-grams used; (**b**) Perplexity for model with 60% n-grams used; (**c**) Perplexity for model with 80% n-grams used.

**Figure 6:**
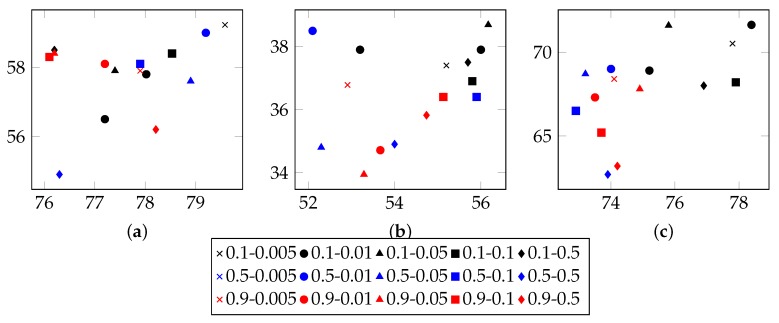
Selection α and β. Correctness-Conciseness scatter diagrams used to compare the quality of inferring (**a**) FTP message formats; (**b**) CoAP message formats; (**c**) XMPP message formats.

Several parameters affect the performance of LDA:

1. I, the number of iterations.
  Theoretically, with increasing times of iterations, the best parameters can be sampled. However, for specific model, it converges after appropriate iterations. Extra iterations can barely improve model but bring more computational cost [24,25].
  In order to find an appropriate iteration count, this approach set types number from 20 to 80 and used 40%, 60% and 80% n-grams to infer LDA parameters. Figure 3, Figure 4 and Figure 5 illustrate the basic results for selecting I, i.e., the number of iterations. For FTP, it could typically reach the optimal point by about 5500 iterations. For CoAP, it needed 8500 iterations to converge. For XMPP, this value was 8000 iterations. According to these results, this approach set the iterations counts to be 6500, 9500, 9000 for FTP, CoAP and XMPP, respectively. By the extra 1000 iterations, this study made sure that the model can converge.
2. N, the proportion of n-grams to be used.
  In the case of thousands of messages, they might generate numerous n-grams, some of which were useless. With a limited number of n-grams, e.g., 40% in the experiments, the model failed to classify messages into different types, since n-grams are too few to represent each type. However, with too many n-grams, the model might consider extra unimportant n-grams, which would also affect the accuracy. We observed that when using 60% n-grams, the model generally performed well, except for conditions with small type counts. As a result, this approach used 60% n-grams, although perplexity for experiments using 40% n-grams are generally lower. The selected 60% n-grams were obtained by filtering others according to TF-IDF scores.
3. T, the types number of messages to be used.
  The number of messages types determines the granularity of message clustering. With an increasing number of types, the model achieves fine-grained results. However, this also brings extra computational cost. Figure 3, Figure 4 and Figure 5 also illustrate the optimal values of T. For FTP, when using 60% n-grams, the model performed well with T = 65. For both CoAP and XMPP, this value was 50.
4. The hyper parameters that controls two Dirichlet distribution, i.e., α and β.
  In this approach, the distribution controlled by α is per-message type, and the distribution controlled by β is per-type n-gram. With I, N and T fixed, i.e., iteration counts, proportion of n-grams and types number of message, it was not hard to decide the optimal values for α and β.
  Original LDA sets 50/K as default α value, where K is the number of topics. With α<1, the distribution tends to be normalized and for a document, most topics will hold low probability. Considering this, 0.1, 0.5 and 0.9 were used as candidates for α.
  Practically, models tend to employ small values for β, e.g., 200/W, where W is the number of words. Considering the large scale of n-grams, this study did not use 200/W to calculate an experience value. Instead, it tested 0.01, 0.05, 0.1, 0.2, and 0.5 for β and used the optimal one as experiment parameter.
  Figure 6 demonstrates the basic results of tuning these two parameters. By considering both Correctness and Conciseness, this study used Correctness-Conciseness scatter diagrams to compare the performance for varying α and β values. For the mark “a-b”, “a” means value of α and “b” means value of β. The upper-right points correspond to optimal parameters.
  According to experimental results, for FTP, this study selected 0.1 and 0.005 for hyper parameters α and β, respectively. For CoAP, 0.1 and 0.05 were used. For XMPP, 0.1 and 0.01 were used. The formats of XMPP are based on XML, which makes the conciseness for XMPP better, whereas for CoAP, some fields are bit-aware, which makes it confused to classify a message into some type. This thus, explains the lower conciseness for CoAP. By tuning these two hyper parameters, relatively optimal values were selected for final experiment.

### 5.4. Performance Evaluation in Terms of Correctness and Conciseness

**Figure 7:**
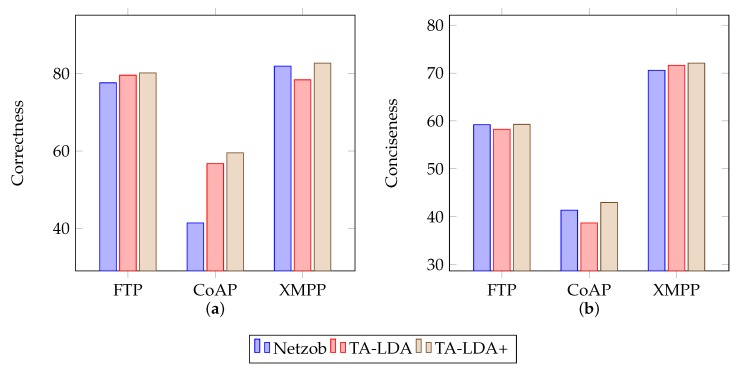
Correctness and Conciseness Comparison. (**a**) Correctness for different approaches applied to infer message formats; (**b**) Conciseness for different approaches applied to infer message formats.

The experiments in Netzob generally proved the effectiveness of this approach, as illustrated in Figure 7, where “Netzob” means the original framework with manually selected delimiters, “TA-LDA” means the basic version of this approach and “TA-LDA+” means this approach enhanced with delimiters selected manually.

Table 1 demonstrates the general improvements for correctness and conciseness by hiring the proposed approach in Netzob.

For correctness, Netzob was improved by replacing clusterByAlignment with clusterByLDA. During experiments in original Netzob, this study manually set “<”, “>” and “=” to be delimiters. This makes Netzob a little better than the basic version of the proposed approach but it is not so automatic. However, the general results show the effectiveness of the proposed approach. With proposed clustering approach, i.e., clusterByLDA, Netzob performed better. This could be explained by the use of the higher-level type information instead of literal bytes data.

For conciseness, the improvements are not so obvious. Without assigning delimiters, this approach performed slightly worse for CoAP and HTTP. For these two protocols, their formats contain more information and need more accurate inference. However, the slightly worse performance is a trade-off between performance and automation. This shows that the proposed approach improves Netzob’s automation with only slight performance reduction.

## 6. Conclusions

This study develops a type-aware approach to message clustering. The approach can extract latent type information hidden in protocol messages and utilize it to cluster messages.

The approach utilizes a variant of LDA to characterize protocol messages with their types and n-grams. Type information is represented as type distribution of LDA. This distribution is used as distance vector for each message, which guides message clustering. This approach ensures that messages are clustered according to type information, the higher-level semantic information for messages.

Experimental results indicates that the results of message clustering are improved using proposed type information. The results also demonstrate that the correctness and conciseness are significantly improved, e.g., the figures are 43.86% and 3.87%, respectively, for CoAP protocol.

Furthermore, determination of parameters can affect the results. Experiments were conducted to set optimal parameters, e.g., the number of iterations, the number of types, and Dirichlet prior parameters.

The approach holds the potential to improve the extraction of message formats as well as state machines. Overall, the work paves the way to quantitatively expressing and utilizing type information for protocol messages by hiring topic generative models.

## Figures and Tables

**Table 1:** Improvements for Netzob.

	FTP	CoAP	XMPP
Improvements for Correctness	3.28%	43.86%	0.95%
Improvements for Conciseness	0.10%	3.87%	2.15%
